# Confirmation of the ScanPyramids North Face Corridor in the Great Pyramid of Giza using multi-modal image fusion from three non-destructive testing techniques

**DOI:** 10.1038/s41598-025-91115-8

**Published:** 2025-03-18

**Authors:** Thomas Schumacher, Polina Pugacheva, Hussien Allam, Alejandro Ramirez-Pinero, Benedikt Maier, Johannes Rupfle, Khalid Helal, Olga Popovych, Amr G. Hamza, Mohamed Sholqamy, Mohamed Fath-Elbab, Mohamed Elkarmoty, Mehdi Tayoubi, Hany Helal, Christian U. Grosse

**Affiliations:** 1 Civil and Environmental Engineering, Portland State University, 1930 SW 4th Avenue, Portland, OR 97201 USA; 2 Chair of Non-Destructive Testing, TUM School of Engineering and Design, Technical University of Munich, Franz-Langinger-Str. 10, 81245 Munich, Bavaria Germany; 3 Rock Engineering Laboratory, Faculty of Engineering, Cairo University, Gamaa Street 1, Giza, 12613 Egypt; 4 UNESCO Chair On Science and Technology for Cultural Heritage, Faculty of Engineering, Cairo University, Gamaa Street 1, Giza, 12613 Egypt; 5 Department of Mining, Petroleum, and Metallurgical Engineering, Faculty of Engineering, Cairo University, Gamaa Street 1, Giza, 12613 Egypt; 6 Dassault Systèmes, 10 Rue Marcel Dassault, 78140 Vélizy-Villacoublay, France; 7 Heritage Innovation Preservation Institute (HIP Institute), 50 Rue de Rome, 75008 Paris, France

**Keywords:** Archeology, Cultural heritage structures, The Great Pyramid of Giza (Khufu’s Pyramid), ScanPyramids North Face Corridor, Non-destructive testing, Multimodal image fusion, Geophysics, Civil engineering

## Abstract

While non-destructive testing (NDT) measurements have been reported individually for archeological surveys of cultural heritage structures, only a few studies to date have attempted to combine NDT images by means of image fusion (IF). In this article, novel multimodal IF results from three different NDT techniques collected at the Chevron located on the Great Pyramid of Giza (aka. as Khufu’s Pyramid) are presented. The Chevron is an assembly of limestone blocks located in front of the recently confirmed ScanPyramids North Face Corridor (SP-NFC), which had been previously hidden for 4500 years. Under the research activities of the ScanPyramids mission, three profiles located on the Chevron were selected to explain multimodal IF in detail and highlight its usefulness in archeology. The NDT techniques employed in this study include ground penetrating radar (GPR), ultrasonic testing (UST), and electrical resistivity tomography (ERT). A discrete wavelet transform (DWT)-based algorithm was employed to merge the reconstructed images from the three techniques for each profile, producing a single composite image. The final fused images contain pertinent information from all modalities, allowing to validate assumptions used to create the individual reconstructed images, and enable a more detailed examination of some of the conclusions reached in the authors’ previous ScanPyramids work.

## Introduction

### Background

The objective of image fusion (IF) is the process of combining multiple input images into a single composite image^1^. The fused image is intended to contain pertinent information from the individual input images and is more useful for human expert or machine perception. IF is a well-established data processing technique in the medical field, see e.g., Hermessi, Mourali et al.^2^ and remote sensing, see, e.g., Ghassemian^3^ and Pohl and Van Genderen^4^ respectively. IF has also found some limited interest in civil engineering, in particular in imaging of concrete structures using ground penetrating radar (GPR) and ultrasonic testing (UST) images^5–7^. It should be noted that IF is a subset of data fusion^8^, in that it uses processed data in the form of reconstructed images rather than raw data in the form of individual signals. In this work, signal-level fusion would not be possible because it requires the measurement points of each technique to match, which is impractical. For example, the locations where individual GPR and UST waveforms are collected are not the same, and selecting common points would dramatically reduce available GPR waveforms, which in turn would reduce the resolution of the reconstructed images. Moreover, ERT measurements are not compatible with GPR and UST waveform data, and image reconstruction is thus required before any sort of fusion can be employed.

A recent review of close-range sensing and data fusion methods for cultural heritage structures, in which the authors include GPR and UST, is provided by Adamopoulos and Rinaudo^9^. It should be noted that the direct fusion of GPR and UST images is not reported, but rather each of them individually with techniques such as laser scanning and photogrammetry. Hess, Petrovic et al.^10^ describe the use of light detection and ranging (LiDAR) and structure from motion (SFM), as well as combining air- and ground-borne measurements, for documenting and diagnosing cultural heritage sites using surface observations. With respect to the application of multimodal IF to cultural heritage structures using NDT-based images, very little literature exists. Salazar, Gosalbez et al.^11^ proposed the fusion of GPR and UST tomographs to characterize historic walls. In 2013 the same group reported a study where they added impact echo data to perform three-way fusion^12^. Note that tomography requires access to more than one side of an investigated member, which is often not possible, particularly in large cultural heritage structures. In studies on a larger scale in the Campana region of Greece, Karamitrou, Bogiatzis and Tsokas^13^ used IF of magnetic and electrical resistivity images with prior information about the orientation of archaeological structures, to improve their detectability. The most recent application of IF in the field of cultural heritage structures, and directly related to the work discussed in this article, is documented in Elkarmoty, Rupfle et al.^14^, which introduced IF using GPR and UST images. The work presented in this article is a novel extension, as it integrates a third NDT technique, electrical resistivity tomography (ERT), to study the internal structure of Khufu’s Pyramid.

In this article, a novel multimodal IF application is presented that uses 2D reconstructed images from three different NDT techniques, namely GPR, UST, and ERT, to confirm and examine a previously hidden void in Khufu’s Pyramid: The ScanPyramids North Face Corridor (SP-NFC).

### Motivation

Combining several NDT techniques to examine a structure offers multiple advantages. First, each technique is based on a different physics principle (i.e., different modality), and thus has different detection capabilities and limitations. Second, using techniques that have some overlapping capabilities can help confirm observations and measurements of each technique. Third, variables used to create the individual input images such as wave velocity can be validated if the reflectors appear in the same location, which is enabled using IF. The objective of this article is to demonstrate the applicability and usefulness of multimodal IF for application to cultural heritage structures. This is achieved by showing new results, which are based on multimodal IF using reconstructed images from GPR, UST, and ERT, allowing further confirmation and the revealing of additional details of the recently discovered SP-NFC.

### Measurement locations

**Fig. 1:**
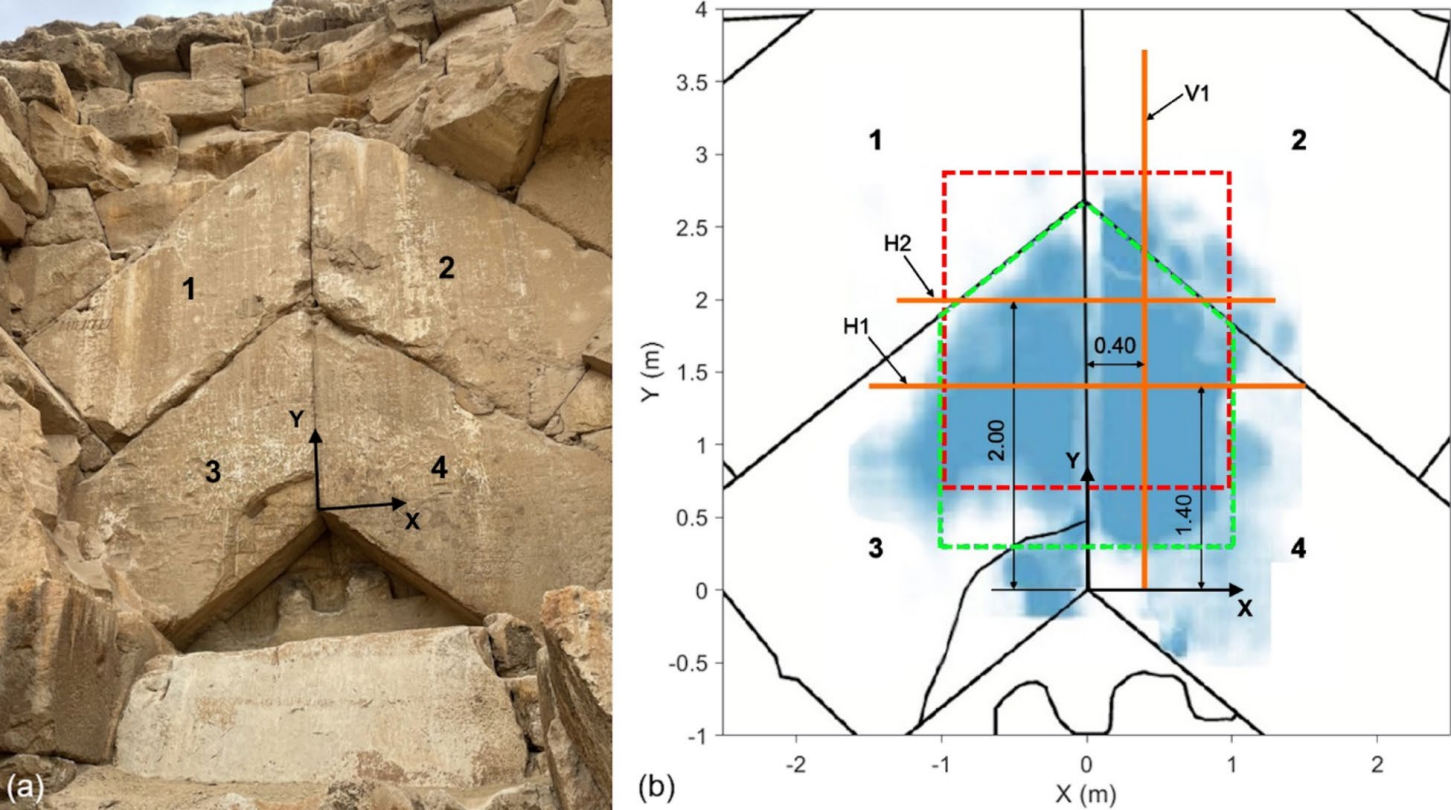
The Chevron located on the north face of Khufu’s Pyramid: (**a**) Photo and (**b**) sketch of elevation view with location of IF profiles (orange lines) discussed in this article. The red and green dashed lines in (**b**) show the estimated cross-sections of the SP-NFC as they are reported in Procureur, Morishima et al.^15^ and Elkarmoty, Rupfle et al.^14^, respectively. The blue reflector shown in (**b**) represents the backwall of the Chevron blocks and is from a reconstructed depth slice image from GPR measurements (200 MHz antenna) reported in Elkarmoty, Rupfle et al.^14^.

The Chevron on Khufu’s Pyramid is an assembly of four large limestone blocks that are arranged in an inverted double ‘V’ pattern. Figure 1 shows (a) a photo and (b) a sketch of the elevation view of the Chevron with (b) highlighting the locations of the IF profiles (orange lines) selected and discussed in this article. The blue reflector shown in (b) represents the backwall of the Chevron blocks and is from a reconstructed depth slice image obtained from GPR measurements (200 MHz antenna) reported in Elkarmoty, Rupfle et al.^14^. Darker shades of blue correspond to larger amplitudes of reflection. The depths of the slices were determined based on the B-scan data and were different for each of the blocks. Note that Block 1 did not produce a detectable reflector. The red dashed and green dashed outlines represent the estimated cross-section of the SP-NFC, as predicted by cosmic ray muon tomography^15^ and subsequently confirmed by means of GPR and UST measurements using IF^14^, respectively.

## Measurement techniques

In this chapter, the non-destructive testing (NDT) techniques used to produce the images discussed in this article are briefly introduced. Measurements were performed during three fieldwork campaigns carried out from 2020 to 2022 and are limited to the Chevron blocks located on the north face of Khufu’s Pyramid. A wooden scaffolding allowed measurement access to the upper areas of the Chevron. The strengths and limitations of each NDT technique with respect to the application to the Chevron are also discussed.

### Ground penetrating radar

Ground penetrating radar (GPR) is considered one of the most important non-destructive geophysical techniques used in archaeological sites prospections^16,17^. GPR relies on sending electromagnetic waves that propagate at a certain velocity into a host medium^17^. When these waves encounter any sub-surface anomalies that differ in electromagnetics properties, i.e., have different relative permittivities, from the host medium, a portion of the wave energy is reflected while another portion is refracted and spreads in the new medium at a different velocity. The reflected waves are received by an antenna, or an array of antennas, housed in the instrument. Based on the travel time of the reflected waves, and by assuming a wave velocity, the depth of anomalies located under the surface are determined.

**Fig. 2:**
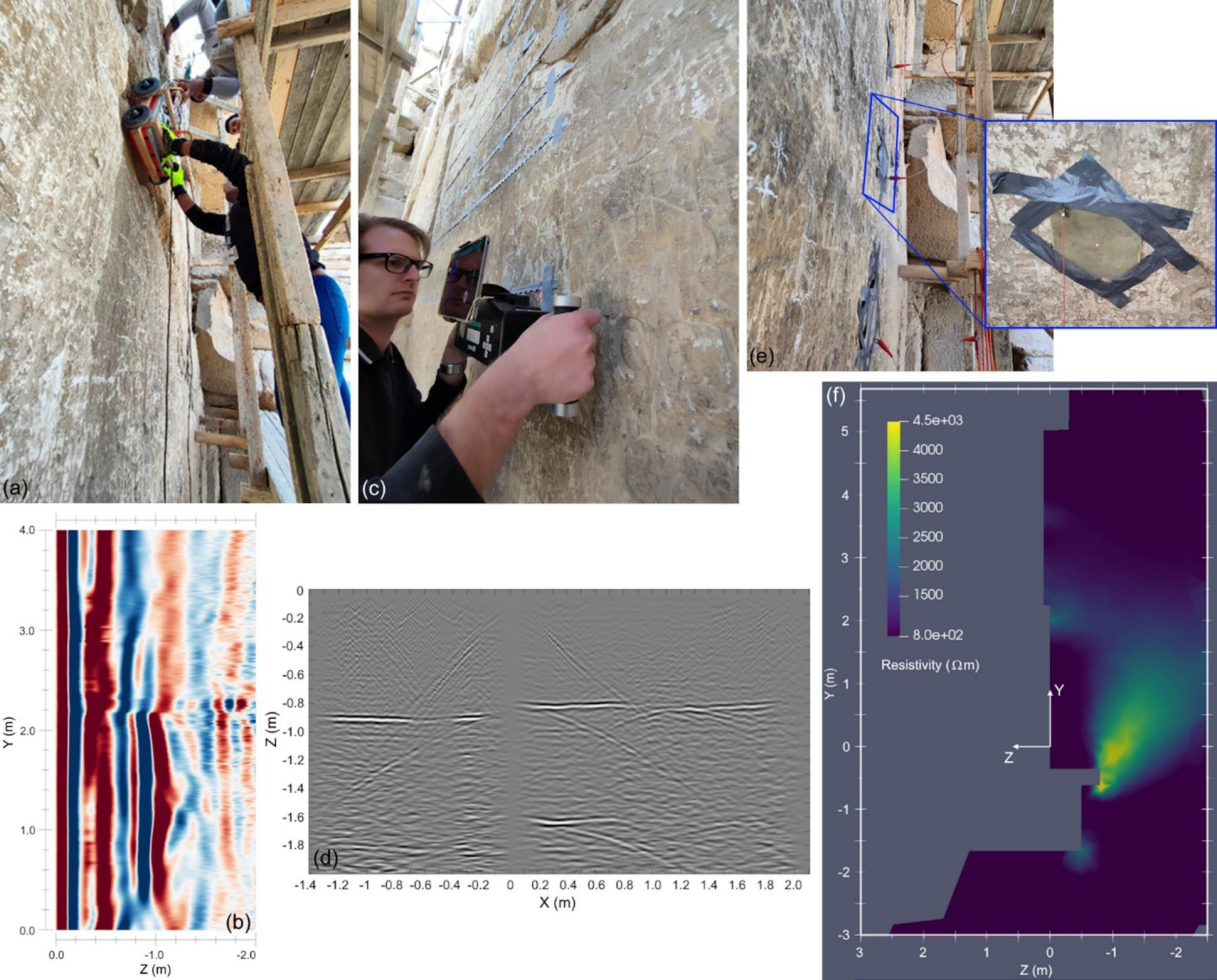
Photos and sample reconstructed images from the three NDT techniques used in this work: GPR—(**a**) Photo of ongoing vertical measurement with ground-coupled instrument and (**b**) sample reconstructed GPR image (200 MHz antenna) for vertical profile V1 located at X = 0.4 m; UST—(**c**) Photo of ongoing horizontal measurement with ultrasonic shear-wave pulse echo array instrument and (**d**) sample reconstructed UST image for horizontal profile H1 located at Y = 1.4 m; ERT—(**e**) Photo of electrodes arranged vertically on a straight line and (**f**) sample reconstructed ERT slice from a 3D model for vertical profile V1 located at X = 0.4 m. For profile locations see Fig. 1b. A collection of all reconstructed images can be found in the Appendix, Figures A1 through A3.

GPR measurements on the Chevron were conducted with six different antennas offering a frequency range of 200 to 800 MHz^14^. The measurements associated with the profiles discussed in this article were collected using GPR instruments equipped with dual frequency antennas with 300/800 MHz (GSSI, Model 50300/800) and 200/600 MHz (Hi-Mod IDS GeoRadar). The grid spacing for the GPR measurement profiles varied from 50 to 200 mm. Details about the measurements and the image reconstruction algorithms used can be found in Elkarmoty, Rupfle et al.^14^ and Elkarmoty, Helal et al.^18^. A customized wooden cart and wooden bars were used to ease the movement of the antennas over the Chevron surface and ensure that the scanned profiles were horizontal and vertical (see Fig. 2a).

GPR has proven to be a reliable technique for blocky structures^14^. However, the attenuation of the electromagnetic waves in this medium is higher compared to continuum structures, which limited the penetration depth for all the antennas used, but in particular for the ones with the higher frequencies. The estimated mean wave velocity for limestone blocks at this site that were accessible from two sides considering the depth-to-known-reflector (DTKR) approach was determined to be 0.112 m/ns. Standard processing steps were performed using the software package Reflexw©^19^, including applying a third-order Butterworth bandpass filter, time-zero correction, application of a gain function with linear and exponential components, and background removal that subtracts the average trace. Images were reconstructed using the synthetic aperture focusing technique (SAFT), which was also done in Reflexw©. Details about the measurements, additional post-processing, and the used image reconstruction algorithms can be found in Elkarmoty, Rufle et al.^14^ and Elkarmoty, Helal et al.^18^. Figure 2b shows a sample reconstructed GPR image located at X = 0.4 m (= vertical profile).

### Ultrasonic testing

In ultrasonic testing (UST), elastic waves are emitted into a material and reflected at interfaces between materials. The amount of energy reflected at such a boundary is high if the acoustic impedance (or reflectivity of the boundary materials) is different^20^. These impedance values are several orders of magnitude higher for the boundary between rock and air than between rock and metal. This makes ultrasound to be the ideal modality to detect voids within blocks or boundaries between them. However, since most of the energy is reflected at an air boundary such as an open joint, no, or little information can be obtained about the region behind it. For the UST measurements on the Chevron, an ultrasonic eight-channel shear-wave pulse echo array was used (Screening Eagle Technologies, Model Pundit PD8000), which is shown in Fig. 2c. This instrument has eight channels, i.e., *n* = 8, three transducers per channel. Signals from the three transducers per channel are averaged. Consistent coupling of the spring-loaded point-contact shear wave transducers occurs via dry-coupling when the instrument is pushed against the surface. The measurement process at each position is as follows: One-by-one, each channel serves as the pulse emitter, while all rows to the right of the emitting channel record the response. This produces a total of ${{(n)(n - 1)} \mathord{\left/ {\vphantom {{(n)(n - 1)} 2}} \right. \kern-0pt} 2} = 28$ ultrasonic waveforms per measurement position. Note that two of these instruments can be combined to form a 16-channel unit, which produces 120 measurements. Results from this configuration will be reported elsewhere. After a sequence is completed, the instrument is moved to the next measurement position. Typically, a measurement increment of 100 mm was used in this work, allowing sufficient overlap of measurements. The instrument was set to the lowest selectable pulse frequency, which was 25 kHz, allowing for a sensible compromise between spatial resolution and penetration depth.

UST images were reconstructed using the software InterSAFT^21,22^. This is possible due to the array configuration of the instrument, and thus different compared to how GPR data is collected. Typical shear wave velocities measured on the Chevron blocks determined using the array’s built-in calibration function range from approximately 1300 to 2200 m/s. This wide range can be explained by the differences in surface deterioration of the limestone, which in some cases represented a challenge for performing UST measurements. The final mean shear wave velocity used for Block 3 was 1800 m/s and for Blocks 2 and 4, a value of 2040 m/s was used. These shear wave velocities produced reflector depths consistent with the ones observable in the GPR images and as was confirmed in one location that was accessible in one location from both sides. The grid spacing for the UST measurement profiles varied from 100 to 200 mm. Details about the measurements and the image reconstruction algorithms used can be found in Elkarmoty, Rupfle et al.^14^ and Elkarmoty, Helal et al.^18^. A sample reconstructed UST image that was collected at Y = 1.40 m (= horizontal profile) is shown in Fig. 2d.

### Electrical resistivity tomography

Electrical resistivity tomography (ERT) is a ground-based geophysical technique that employs a current source to investigate the electrical properties of the subsurface. In electrical resistivity measurements, different electrode pairs supply current and measure potentials. Calculated following Ohm’s law, resistance is converted into apparent resistivity by accounting for a geometric factor. Subsequently, apparent resistivity values are used to reconstruct true resistivity distribution during an iterative inversion process ^23^. For this work, special non-invasive sheet electrodes made of stainless steel were used. Galvanic contact with the blocks’ surfaces was achieved using a sponge moistened with freshwater. The measurements were carried out using an ABEM Terrameter LS2 instrument. A photo of the measurement setup is shown in Fig. 2e with the inset showing a close-up view of an individual electrode. The data were measured using a dipole–dipole configuration along ten parallel profiles spaced at 0.25 m. This data collection method makes it possible to implement a 3D approach to data processing. The spatial resolution of the ERT investigation depends on the density of the measurements, severity of resistivity contrasts in the study area, and the choice of inversion parameters, particularly, mesh size and degree of regularization^24^.

A simplified 3D model was created and discretized into tetrahedral elements using the software Gmsh^25^ for effective finite element (FE) modeling of the Chevron with its geometrically complex shape. The data were inverted using the open-source software pyGIMLi^26^. The inversion is based on the Gauss–Newton method with global regularization, in which the data points are weighted with their measurement errors. To obtain a clear image of the anomaly and to reconstruct its shape and boundaries most accurately, robust inverse solution L1 was applied within error limits based on the measurement reciprocity errors. The reconstructed images represent vertical, horizontal, and depth sections derived from a 3D model and converted from cell data to point data format using the Paraview software^27^. Details about the measurements and inversion results will be published in upcoming article about the application of the ERT technique on the Chevron zone. Figure 2f shows a sample reconstructed ERT image located at X = 0.4 m (= vertical profile).

## Methodology

Two-dimensional (2D) reconstructed images from three different NDT techniques were available and used in this study: Ground penetrating radar (GPR), ultrasonic testing (UST), and electrical resistivity tomography (ERT). The first and second were created using synthetic aperture focusing technique (SAFT) and total focusing method (TFM) algorithms, respectively, and the third one using tomographic reconstruction. Technically, the GPR and UST-based images are different representations compared to the ERT images. The former two represent images showing internal or (sub-surface) reflectors, highlighting the interfaces between two materials causing the wave energy to be reflected. The third one is a tomographic slice obtained from the 3D-inverted model of electrical resistivity data. The advantage of ERT is that it provides volumetric information, which GPR and UST cannot. As such, ERT can confirm that a reflector visible in the GPR and UST images is not due to a thin air layer between blocks, e.g., a joint, but is caused by an air void of significant depth. Image fusion (IF) was deemed ideal for combining pertinent information from these three modalities.

**Fig. 3:**
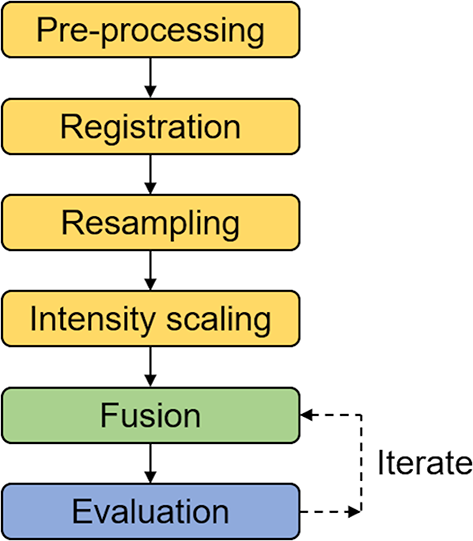
Illustration of methodology followed in this study. Numbering refers to subsequent sections, which discuss each individual step in detail.

A pixel-level spatial domain discrete wavelet transform (DWT)-based fusion algorithm^1^ was employed for this study. Before the input images (= individual reconstructed GPR, UST, and ERT images) can be fused, they need to be registered, sized, and scaled, so that they align properly. Generally, registration involves spatially aligning two images by transforming one of the images (= input image) to match another image (= reference image) using geometric spatial transformations, i.e., translation, rotation, and scaling, of the input image^1^. The methodology followed in this study are illustrated in Fig. 3 and are explained in detail in Sections 3.1 through 3.7.

### Pre-processing

This step involves loading the input images into the coding environment. It is assumed that the resolution of these images is sufficiently high; a discussion regarding minimum resolution requirements is presented in Sect.  3.3. In this study, the input images are the individual reconstructed GPR and UST images, which are SAFT/TFM-based images, as well as the ERT image, which is a tomographic image. All images were defined as 8-bit three-component images in their original resolution. Once imported, an image is defined as a three-dimensional matrix with dimensions, [*n* x *m* × 3]^28^. The GPR and UST images were defined in grayscale and the ERT image in pseudo-color. Keeping some input images in grayscale and some in color is a common approach in the field of medical PET/MR imaging^1^. In this study, the GPR and UST images provide details about the reflectors, which can be represented in grayscale, while the ERT image shows the spatial distribution of resistivity, which is most effectively represented using colors. The color map for resistivity selected in this study is Parula, which shows low and high resistivities in blue and yellow, respectively. Figure 2f shows an example of an ERT tomographic map with resistivity values.

### Registration

Image registration is performed to ensure that salient features in each input image are spatially and temporally aligned, i.e., they lie in the same orientation and position and are taken at the same time, respectively. In the medical field this is a critical and often complex step and continued subject to research and advancement, especially with the availability of machine learning (ML) techniques^29–34^. Because images from NDT measurements on civil structures have a common coordinate origin and follow pre-defined profiles, registration is typically a straightforward step that can be performed manually. In this study, only a few steps were therefore necessary to register the input images. The GPR images were shifted (= translated) in the depth direction so that the first (direct) wave peak aligned with the surface, i.e. Z = 0. This was done to correctly align the images with respect to depth and ensure that salient features such as reflectors appear in the same location in all input images. Note that the alignment of reflectors between input images also validates the wave velocities used in the imaging algorithms. Additionally, GPR images were shifted by approximately 0.20 m to account for a coordinate offset in the measurement direction. This shift was determined manually using the block joint locations as reference points. No translation was necessary for the UST and ERT images. Finally, image cropping was necessary because the profile lengths as well as the maximum reconstructed depths were different for each modality. While the profile lengths were different for each of the three selected profiles, the depth was set to Z = − 2.0 m for all of them.

### Resampling

This step ensures that each input image has the same dimensions, i.e., *n* x *m* × 3 pixels (and same pixel size, i.e., mm/pixel), which is a prerequisite for the selected image fusion algorithm to work. When resampling images, it is essential that proper interpolation methods are employed^28^. For downsampling, which reduces the resolution of the image, appropriate low-pass filters need to be used prior to resampling, to avoid aliasing effects. For upsampling, which increases the resolution of the image, interpolation methods such as bicubic interpolation can be used. In this case, the output image should be visually checked for artefacts such as Moiré patterns. MATLAB’s imresize function is an appropriate choice to perform down- and upsampling of images^35^. In this study, a common image resolution was selected that provides sufficient spatial resolution of the reflectors in the images. The peak pulse frequencies for GPR and UST measurements were 300 MHz and 25 kHz, respectively, and average wave velocities based on in situ calibration were 112 × 10^6^ m/s and 2000 m/s, respectively. These values result in wavelengths, *λ*_GPR,300_ = wave velocity/frequency = 112 × 10^6^ m/s/300 × 10^6^ Hz = 370 mm and *λ*_UST_ = 2000 m/s/25 × 10^3^ Hz = 80 mm. A common pixel size of 2 mm was selected for all images, resulting in 185 and 40 samples per wavelength for GPR (300 MHz antenna), and UST, respectively, which are both well above the Nyquist criterion. Note that the GPR images from the 200 MHz antenna automatically pass since their wavelengths are three times as large as the ones from the 300 MHz antenna.

### Intensity scaling

**Fig. 4:**
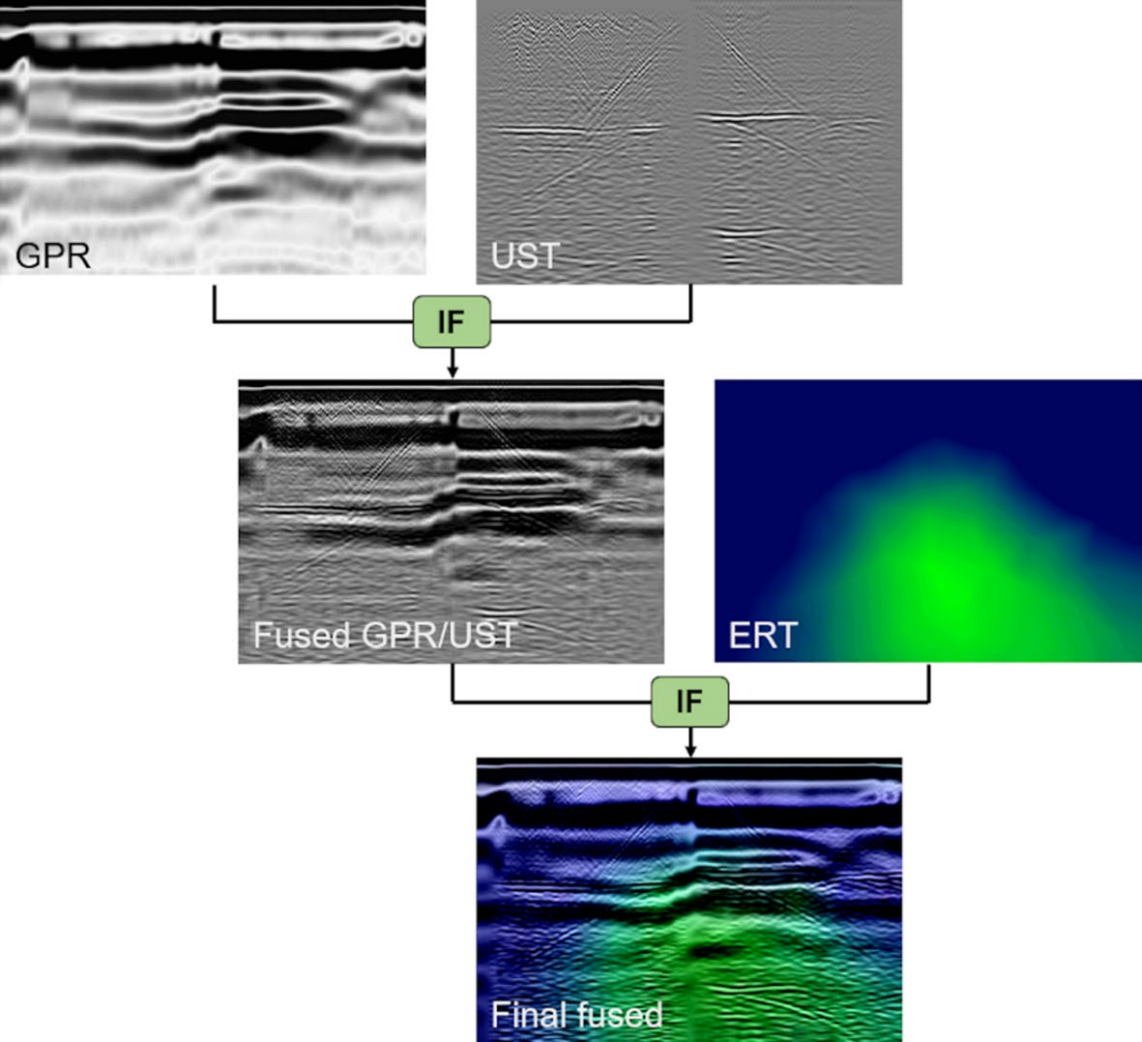
Illustration of multimodal IF process used in this study based on select sample images from horizontal profile H1 (Y = 1.40 m).

Scaling of the pixel intensities can be used to weigh an input image before fusion takes place. Normalizing the intensities puts equal weight on each image. For all images in this work, the range of pixel intensity values were normalized, i.e., the range was set from 0 to 1. This step corresponds to a normalization of the amplitudes, which basically assigns the three modalities the same weight (or importance) in the fusion process. Additionally, the MATLAB function imadjust was applied to both the GPR and UST images, which were kept in grayscale^35^. By default, imadjust saturates the bottom and top 1% of all pixel values, which increases the contrast of the image. The intensity of the ERT image, which was kept in color, was adjusted manually, to ensure that the resistivity differences are discernable in the final fused image. Actual resistivity values should thus only be extracted from the original ERT tomography slices (example see Fig. 4d).

### Fusion

DWT-based image fusion (IF) was employed, which decomposes two input images using specified fusion rules into approximations and details coefficients^36^. The wfusimg function available in MATLAB was used in this study^35,37^. The “sym3” wavelet with a wavelet decomposition level of seven was selected for all processing and fusion was performed by taking the minimum and maximum for the approximation and detail coefficients, respectively. These settings were determined iteratively by means of visual inspection and found to produce the most useful images with the highest information content and contrast. Since three input images were used, two separate fusions were necessary. First, the GPR and UST images were fused, since they are both SAFT/TFM-based images that reveal reflectors, creating the “Fused GPR/UST” image (see Fig. 4). Subsequently, this fused image was merged with the ERT image, which is a tomographic resistivity slice, to produce the “Final fused” image. Note that the sample images used for Fig. 6 are from Profile H1 (for location see Fig. 1b).

### Evaluation

Performance metrics can be used to quantify the information contained within an image ^5^. Common metrics include standard deviation (overall contrast), entropy (information content, richness), average gradient (intensity change), spatial frequency (sharpness), structural similarity (SSIM) (correlation between two images), etc. These metrics can be used to compare different image fusion rules and algorithms or to compare images. For applications like the one discussed here, fusion results are typically evaluated based on expert opinion using visual inspection. The final fused images should have sufficient contrast to highlight the features of all modalities and be free of artefacts. This was used in this study to determine the quality and usefulness of the final fused images and adjust fusion parameters as presented in Sect.  3.5, as necessary.

### Interpretation

The final step is the interpretation of the final fused images. For this study, this was performed using human intelligence (HI), i.e., by working collaboratively as a multi-expertise team. Progress in artificial intelligence (AI) offers opportunities for AI-guided interpretation, which might be explored in the authors’ future work. The HI-based interpretation of the three profiles of this study is presented in Chapter 4.

## Results and discussion

### Profile H1

**Fig. 5:**
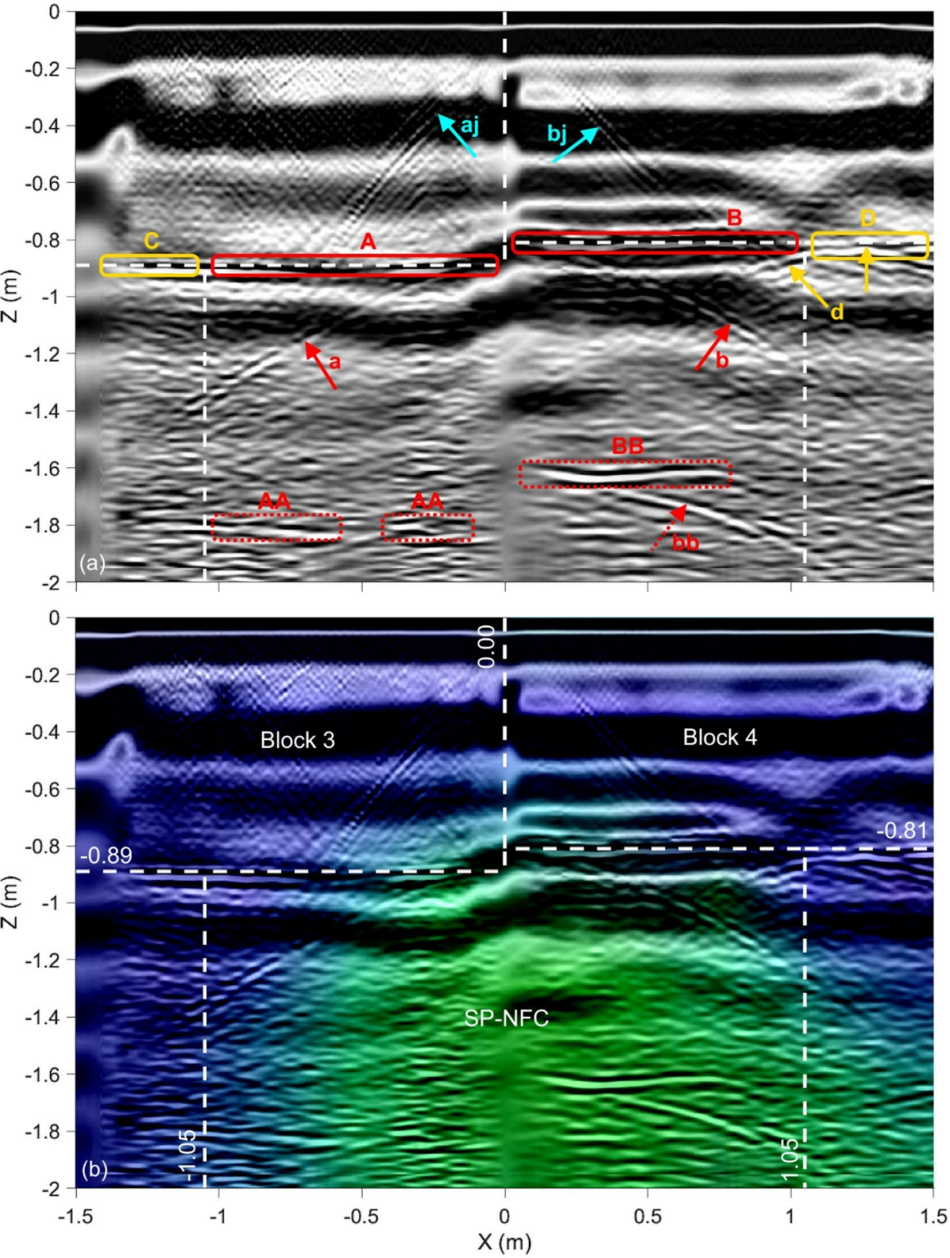
Fused images for Profile H1 (Y = 1.40 m): (**a**) Fused GPR/UST image and (**b**) final fused image. White dashed lines represent previously predicted outlines of the SP-NFC. Red and yellow boxes in (**a**) highlight the main reflectors and extensions beyond previously predicted SP-NFC dimensions, respectively. The input images are shown in Appendix, Figure A1.

Figure 5 shows the final fused image for Profile H1, which is located at Y = 1.40 m (see Fig. 1b). The white dashed lines correspond to the outlines of Blocks 3 and 4 of the Chevron as well as the ScanPyramids North Face Corridor (SP-NFC) walls as they were predicted in ^14^. The three input images along with the two fused images (as illustrated in Fig. 4) are shown in Appendix, Figure A1. Note that the GPR image is based on 200 MHz antenna measurements. In Fig. 5a, the main reflectors “A” and “B” represent the backwall of Blocks 3 and 4, respectively, providing an estimate of the thickness of these blocks. Note that the reflectors depth matches, which validates the wave velocities used to produce the reconstructed images serving as input images. The vertical joint between Blocks 3 and 4 causes the two diagonal reflectors denoted “aj” and “bj”, which originate from the point where the joint meets the outside surface of the two blocks at X = Z = 0.0. The two hyperbolic reflectors denoted “a” and “b” originate from the back corners of the two blocks. Multiples of the main reflectors are also visible, labeled “AA” and “BB”, where “BB” also has a hyperbola, labeled “bb”, which is a multiple of “b”. As can be observed, the main UST reflectors “A” and “B” extend past the previously predicted SP-NFC side walls and are denoted “C” and “D”. The vertical arrow pointing to X ≈ 1.25 m, Y ≈ − 0.81 m in reflector “D” marks the origin of hyperbolic reflector “d”, which can be interpreted as a location where the SP-NFC side wall physically connects to Block 4. Note that a GPR reflection is only visible in “C” but not “D”. The reason for this is that the corners of the side wall blocks are not perfectly square, forming a gap with varying thickness. While most of the ultrasonic wave energy is reflected from a very thin air gap such as a block joint, electromagnetic waves will only reflect from an air gap if it has a thickness comparable to its wavelength. The fact that the corners of the sidewall blocks are not perfectly square can be seen in a video that was published after the announcement of the SP-NFC^38^ (for a screenshot see Fig. 8a). Figure 8b illustrates this observation further and highlights the difference between the two corners (see Details I and II). Figure 5b represents the final fused image, which includes the resistivity distribution from the ERT image, providing concluding evidence that reflectors “A” and “B” are not a result of a thin block joint but due to the presence of a significant air-filled void, i.e., the SP-NFC, located behind Blocks 3 and 4. Note that air has much higher electrical resistivity compared to limestone.

### Profile H2

**Fig. 6:**
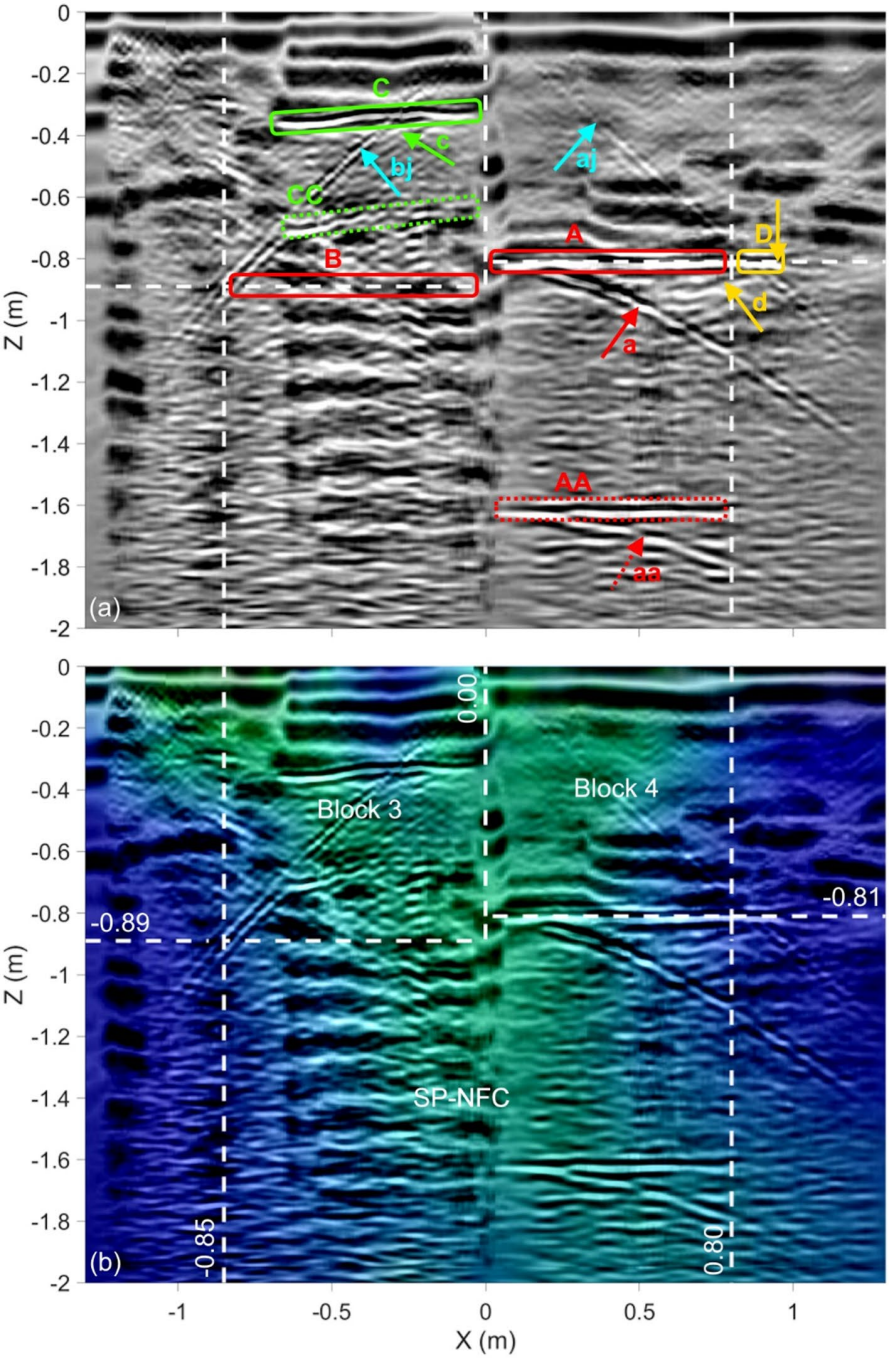
Fused images for Profile H2 (Y = 2.00 m): (**a**) Fused GPR/UST image and (**b**) final fused image. White dashed lines represent previously predicted outlines of the SP-NFC. Red, green, and yellow boxes in (**a**) highlight the main reflectors, the extensions beyond previously predicted SP-NFC dimensions, and the shallow reflector in Block 3, respectively. The input images are shown in Appendix, Figure A2.

Figure 6 shows the final fused image for Profile H2, which is located at Y = 2.00 m (see Fig. 1b). The white dashed lines correspond to the outlines of Blocks 3 and 4 of the Chevron as well as the SP-NFC walls as they were predicted in Elkarmoty, Rupfle et al.^14^. The three input images along with the two fused images are shown in Appendix, Figure A2. Note that the GPR image is based on 300 MHz antenna measurements. In Fig. 8a, the main reflector “A” represents the backwall of Block 4, providing an estimate of the thickness of this block. The vertical joint between Blocks 3 and 4 causes the two diagonal reflectors denoted “aj” and “bj”, which originate from the point where the joint meets the outside surface of the blocks at X = Z = 0.0. The hyperbolic reflector denoted “a” originates from the back corner of Block 4. A multiple of reflector “A” is also visible, labeled “AA” with a multiple hyperbola labeled “bb”. An extension of the reflector past the predicted SP-NFC side wall is denoted “D” with a hyperbolic reflector denoted “d”, which originates at approximately, X = 0.95 m, where the sidewall block connects to Block 4 (marked by vertical arrow). Reflector “B” is associated with the backwall of Block 3, which is only visible in the GPR image. The reason it is not seen in the UST image is the strong shallow reflector denoted “C”, which has a multiple labeled “CC”. The section of Block 3 that lies between the surface the reflector “C” is represented by the region low electrical resistivity. Figure 6b represents the final fused image. Note that the resistivity distribution still shows the SP-NFC but is strongly influenced by the partially opened air-filled block joints.

### Profile V1

**Fig. 7:**
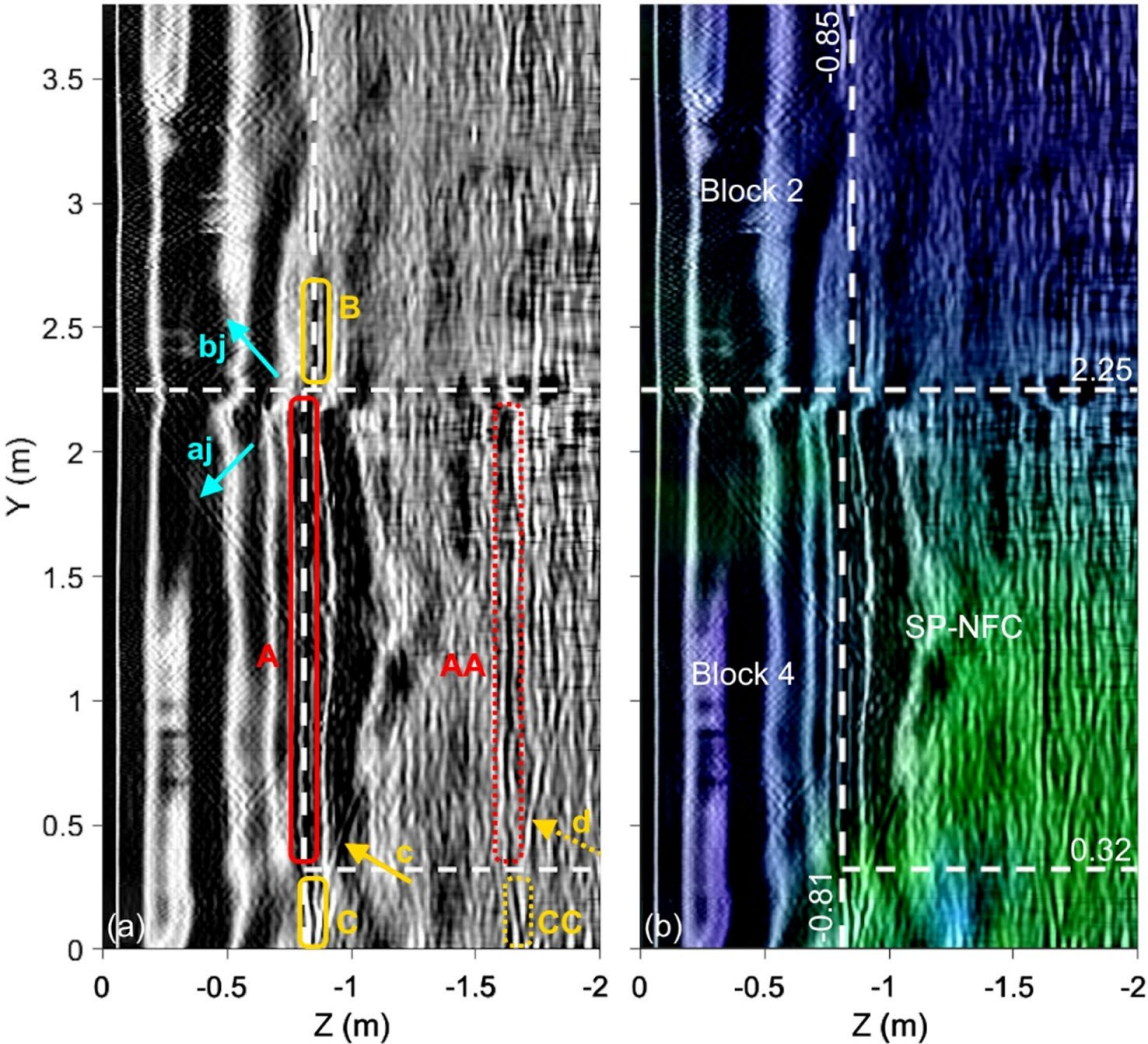
Fused images for Profile V1 (X = 0.40 m): (**a**) Fused GPR/UST image and (**b**) final fused image. White dashed lines represent previously predicted outlines of the SP-NFC. Red and yellow boxes in (**a**) highlight the main reflectors and extensions beyond previously predicted SP-NFC dimensions, respectively. The input images are shown in Appendix, Figure A3.

**Fig. 8:**
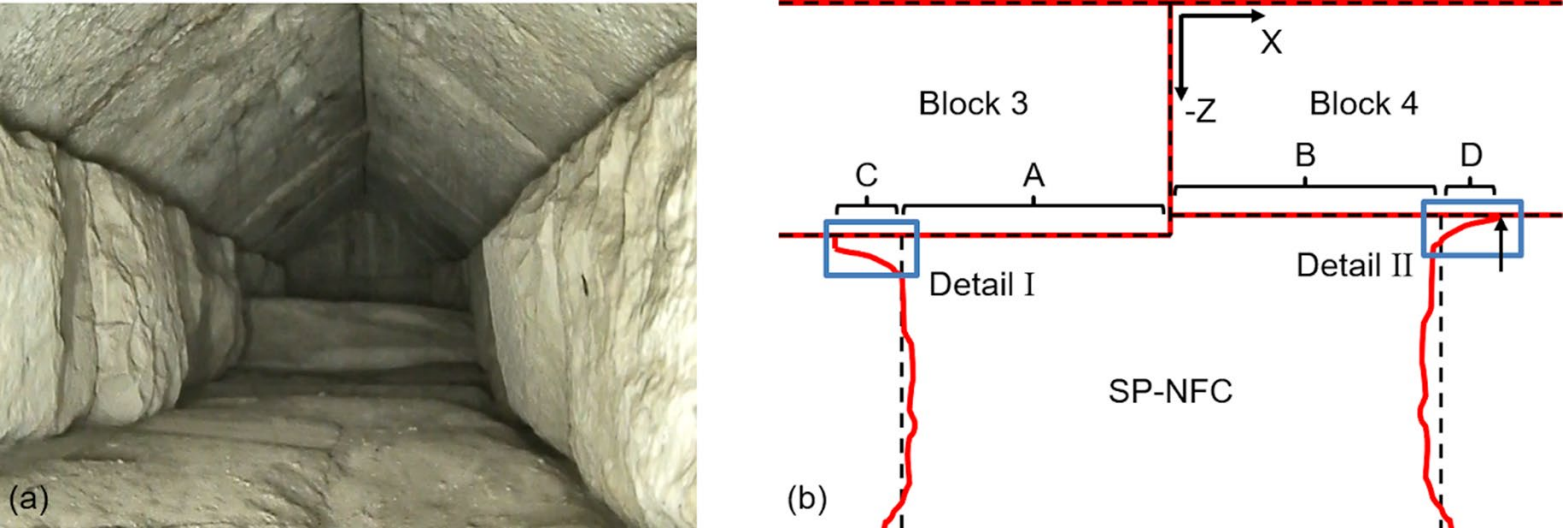
Explanation of results from this study: (**a**) Snapshot from first video taken of the SP-NFC (looking south, -Z direction)^38^ and (**b**) illustration of predicted cross-section at Y ≈ 1.40 m (at the level of Profile H1) of the SP-NFC. Details I and II highlight the possible shape of the blocks’ corners that explain the extended reflectors C and D.

Figure 9 shows the fused image for Profile V1, which is located at X = 0.4 m (see Fig. 1b). The white dashed lines correspond to the outlines of Blocks 4 and 2 of the Chevron as well as the SP-NFC floor and ceiling as they are reported in Elkarmoty, Rupfle et al.^14^. The three input images along with the two fused images are shown in Appendix, Figure A3. Note that the GPR image is based on 200 MHz antenna measurements. In Fig. 7a, the main reflector “A” is the backwall of Block 4 providing an estimate of the thickness of the block. The horizontal joint between Blocks 4 and 2 is indicated by the two diagonal reflectors denoted “aj” and “bj”, which originate from the point where the joint meets the outside surface of the blocks at Y ≈ 2.25 m and Z ≈ 0.0. Extensions denoted “B” and “C” are visible both above and below the main reflector “A”, respectively. Reflector “B” is both visible in the GPR as well as the UST image, which indicates that the roof of the SP-NFC might be higher than originally thought. Reflector “C” has a hyperbola denoted “c”, which originates at approximately, Y = 0.00. Likely, the floor dips down before it connects with Block 4, which is plausible given that it was possible to insert a borescope around X = Y = 0 and Z ≈ -0.81 m to take the video shown in Fig. 8a. In Fig. 7b, it presents the IF result of the three techniques with a clear identification of the location of the SP-NFC corridor as a high-resistive anomaly.

## Summary and conclusions

In this article, a novel application of multimodal image fusion (IF) using measurements from three different NDT techniques, namely ground penetrating radar (GPR), ultrasonic testing (UST), and electrical resistivity tomography (ERT), taken at a select location of the so-called Chevron at Khufu’s Pyramid, is presented. The research described highlights new possibilities for improved data visualization and interpretation based on NDT techniques that are, individually, already used in the field of archeology. Three profiles were selected where reconstructed images from all three modalities were available. A methodology was specifically developed for this study, employing a discrete wavelet transform (DWT)-based algorithm to fuse the images. The following can be concluded from this study:

- The final fused images contain pertinent information from all modalities, which makes interpretation by a human inspector more intuitive. The fact that some reflectors are visible in both the GPR and UST images is useful for the interpretation of the image.
- Reflectors visible in multiple images and at the same locations validate variables used to create the individual images, for example the assumed wave velocities.
- The addition of ERT in this study is novel and adds volumetric information, which was not previously available. The distribution of electrical resistivity allows confidently confirming the presence of the SP-NFC.
- Fig. 8a shows a snapshot of the first video taken from the inside of the SP-NFC in early 2023^38^ and (b) is an illustration of the predicted cross-section of the SP-NFC based on the results from this study at the location of Profile H1. Additional results in the form of 3D visualizations and views of the inside of the SP-NFC based on photogrammetry are documented in^39^. The reflectors extend the point where they were expected to meet the backwalls of Blocks 3 and 4 because of the shape of the side wall block corners, which are highlighted as Details I and II. Note that similar situations are likely the case for the top and bottom corners, which are discussed in Profile V1.

A survey of the SP-NFC using photo cameras and laser-scanning is currently underway. Once it has been completed, the NDT images presented in this study can be confirmed conclusively. Future work includes further refining imaging and IF algorithms and evaluating machine learning for image interpretation.

## Consent to publish

Participants of this study seen in any images have given their permission for publication of the article in an online open access format.

## Supplementary Information

Supplementary Information.

## Data Availability

The datasets used and/or analyzed during the current study are available from the corresponding author on reasonable request.
