# Supplementary material for: Confirmation of the ScanPyramids North Face Corridor in the Great Pyramid of Giza using multi-modal image fusion from three non-destructive testing techniques
Source: Sci Rep. 2025 Mar 18;15:9275. doi: 10.1038/s41598-025-91115-8 (PMC11920056; doi:10.1038/s41598-025-91115-8)
Supplement: Supplementary file 1 — Supplementary Information. [file 41598_2025_91115_MOESM1_ESM.docx]

Supplementary material: Appendix


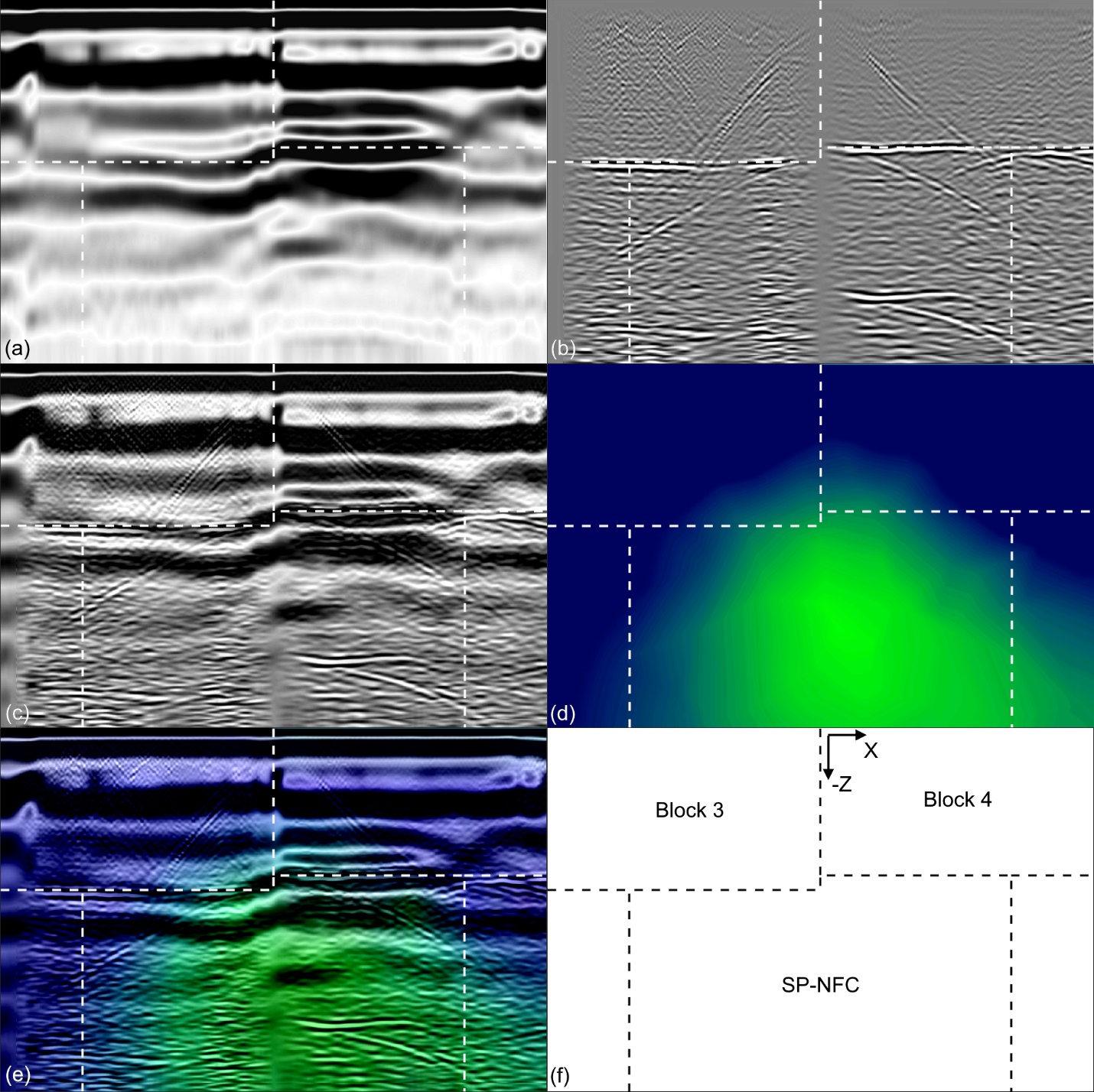


**Figure A1.** Images used for Profile H1 (Y = 1.40 m): (a) Reconstructed GPR image (200 MHz antenna), (b) reconstructed UST image, (c) fused GPR and UST image, (d) reconstructed ERT image, (e) final fused image, and (f) image labels. Common image extents: -1.5 m < X < 1.5 m, 0.0 m > Z > -2.0 m.


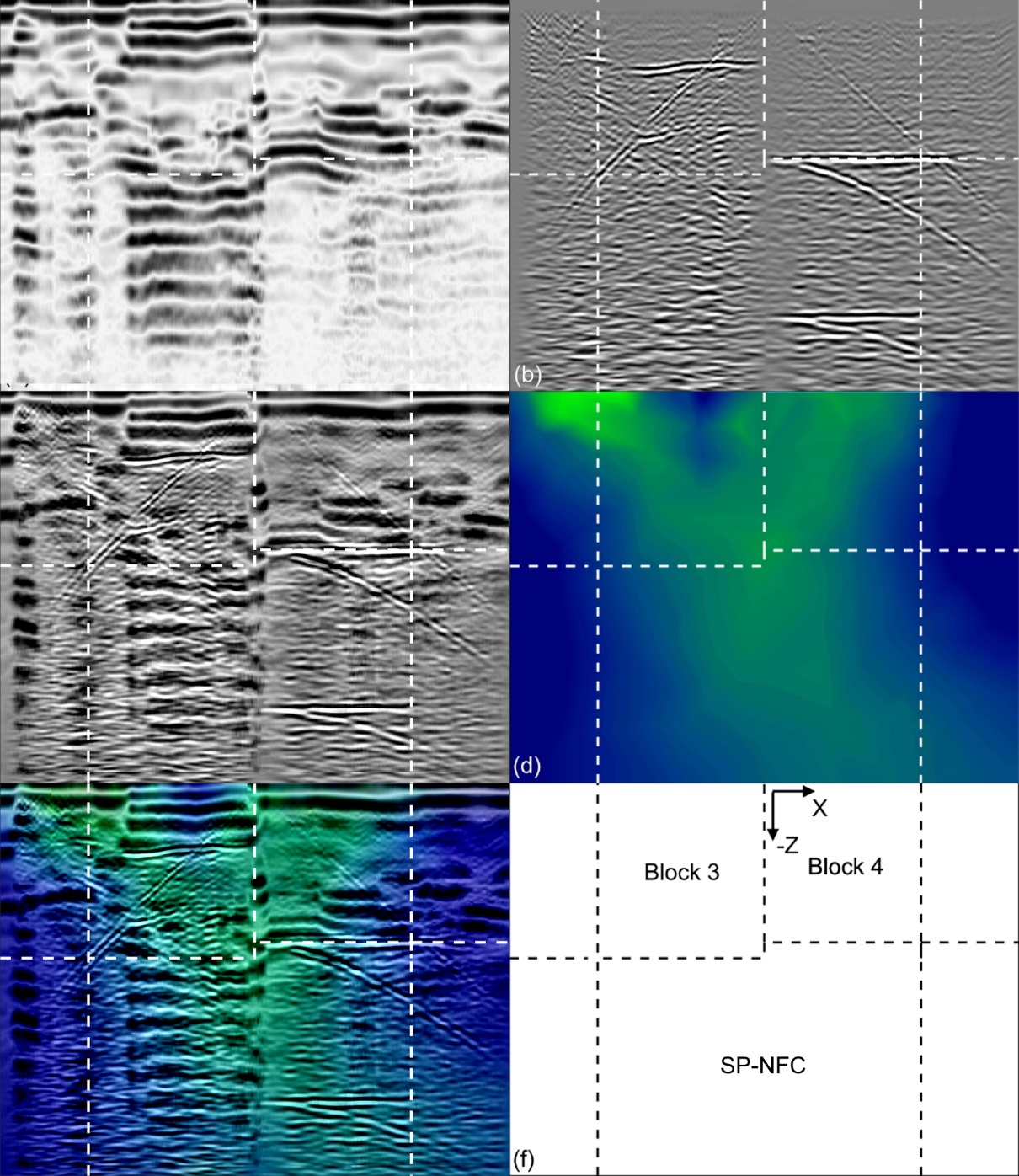


**Figure A2.** Images used for Profile H2 (Y = 2.00 m): (a) Reconstructed GPR image (300 MHz antenna), (b) reconstructed UST image, (c) fused GPR and UST image, (d) reconstructed ERT image, (e) final fused image, and (f) image labels. Common image extents: -1.3 m < X < 1.3 m, 0.0 m > Z > -2.0 m.


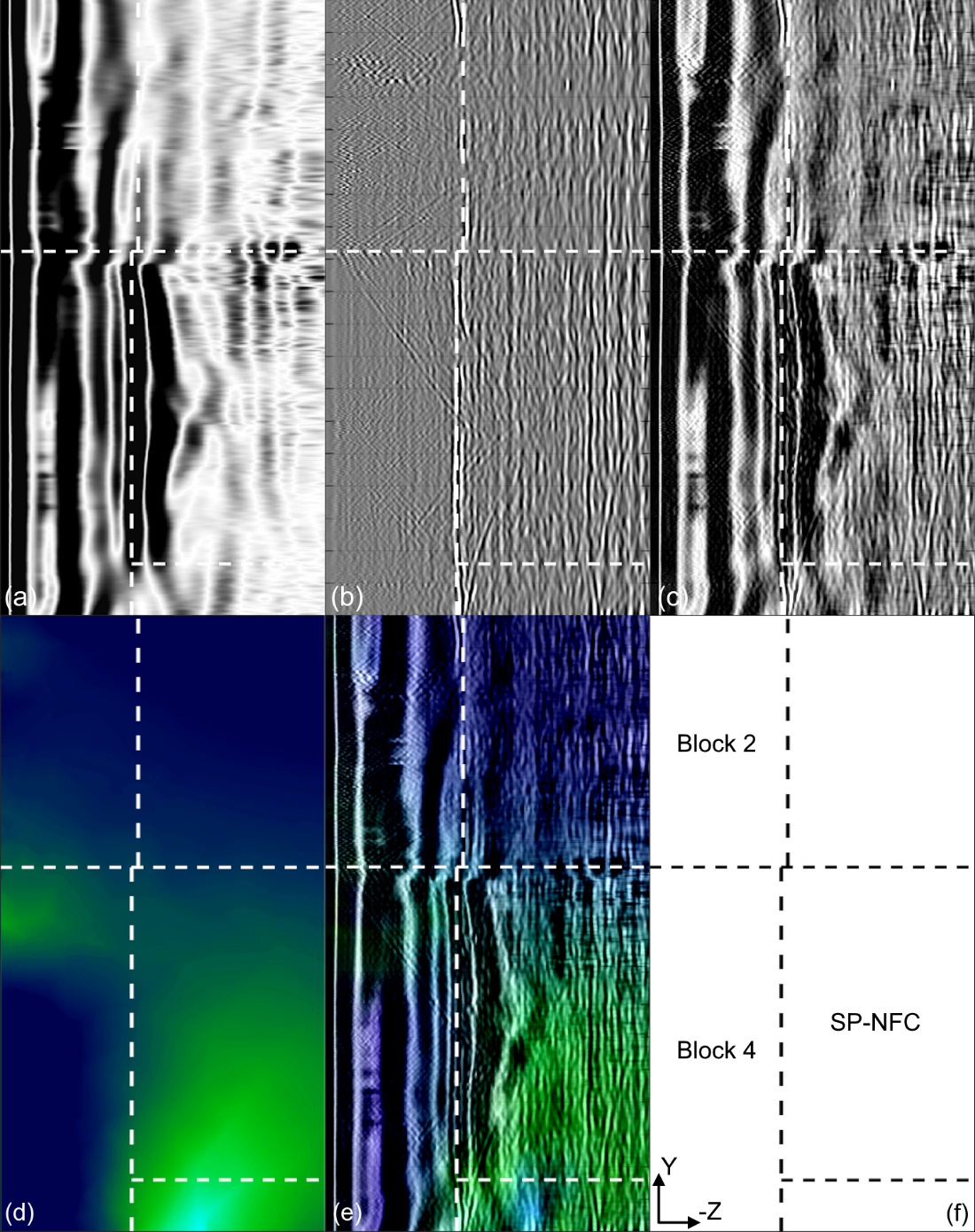


**Figure A3.** Images used for Profile V1 (X = 0.40 m): (a) Reconstructed GPR image (200 MHz antenna), (b) reconstructed UST image, (c) fused GPR and UST image, (d) reconstructed ERT image, and (e) final fused image. Common image extents: 0.0 m < Y < 3.8 m, 0.0 m > Z > -2.0 m.
